# KUAKINI COBRE CONTRIBUTIONS FROM LONG-LIVED OKINAWA PREFECTURE: THE FIRST BLUE ZONE

**DOI:** 10.1093/geroni/igad104.0777

**Published:** 2023-12-21

**Authors:** D Craig Willcox, Richard Allsopp, Makoto Suzuki, Michio Shimabukuro, Moritake Higa, Philip Davy, Bradley Willcox, Trevor Torigoe

**Affiliations:** Okinawa International University, Ginowan, Okinawa, Japan; University of Hawaii, Honolulu, Hawaii, United States; Okinawa Research Center for Longevity Science, urasoe, Okinawa, Japan; Fukushima Medical University, Fukushima, Fukushima, Japan; Tomishiro Central Hospital, Tomigusuku, Okinawa, Japan; Kuakini medical center, Honolulu, Hawaii, United States; Kuakini Medical Center, Honolulu, Hawaii, United States; University of Hawaii, honolulu, Hawaii, United States

## Abstract

The Okinawa-Hawaii connection has a long history. The first group of immigrants to Hawaii from Okinawa arrived in 1899, and descendants of these immigrants make up approximately 20% of the Kuakini Honolulu Heart Program (HHP) cohort. Research in Okinawa, and among the Okinawan community in Hawaii, has provided many insights into the subtle differences in lifestyle and genetics among these populations. In order to gain further insight into the lifestyle and genetic factors associated with healthy aging and longevity, the Kuakini COBRE has built a strong collaborative team of researchers from Okinawa and initiated a number of research projects, as well as cross-Pacific training opportunities for COBRE junior investigators (Research Project Leaders). This presentation will outline new insights into healthy aging and longevity that have been discovered through this collaborative relationship. Our findings highlight the intricate relationship between FOXO3 and telomere length change with age, and include the first validated longevity gene variant that shows an association with negligible loss of telomere length with age in humans. This finding reveals that reduced telomere attrition with age may be a key mechanism for the longevity-promoting effect of the FOXO3 genotype. This presentation will also discuss sex differences that play a role in how, and under what conditions, FOXO3 exerts its role in healthy aging. Finally, future collaborative projects involving mitochondrial health and aging will be introduced.

